# Antibiotic Usage in First Time Coronary Artery Surgery

**DOI:** 10.1186/1749-8090-10-S1-A294

**Published:** 2015-12-16

**Authors:** A Heaney, S Trenfield

**Affiliations:** 1 Royal Brompton Hospital, London, UK

## Introduction

Prophylactic antibiotics have an important role in the prevention of infection following cardiac surgery [1], however, inappropriate or excessive use is associated with the development of antibiotic resistance Cephalosporins, for example, have a well-established association with clostridium difficile [2]. The incidence of nosocomial infections in patients who undergo coronary artery bypass grafting (CABG) is approximately 5% [3]. The current antibiotic regimen for patients who undergo CABG at the Royal Brompton Hospital (RBH) includes cefuroxime 1500 mg at induction and 750 mg prior to sternal closure, followed by two further doses of 750 mg postoperatively, eight hours apart. We conducted a retrospective audit to evaluate adherence to the trust's prescribing guideline for CABG and to review the use of antibiotics in the postoperative period.

## Methods

Patients who underwent first-time CABG from 01.08.2014 to 31.10.2014 were eligible for inclusion. Data were retrospectively collected from the anaesthetic record, the electronic patient record and the medical notes. Data collected included details of antibiotic usage: choice of antibiotic, timing and duration of administration.

## Results

A total of 88 patients were included.

**Table 1:** Antibiotic usage for CABG at RBH.

	Yes	No
Correct antibiotic at induction	86 (98%)	2 (2%)
Antibiotics within 60 minutes of incision	83 (94%)	5 (6%)
Second dose prior to sternal closure	74 (84%)	14 (16%)
Correct postoperative doses	53 (60%)	35 (40%)
Prophlyaxis extended	7 (8%)	81 (92%)
Second-line antibiotics commenced	24 (27%)	64 (73%)

13 (42%) of the 31 patients who received additional antibiotics had some form of positive microbiology, whereas 9 of these patients (29%) had no microbiology analysis. Documentation of the indication for additional antibiotics was only identified for 19 patients (61%).

## Conclusion

This audit found excessive, prolonged use of antibiotics post CABG at RBH. Documentation of the indication for antibiotics was poor.
